# Getting Special Care Dentistry Ready for a Foreseeable Future. Reinstated Services and Mitigation Measures to Curb COVID-19 Disruption

**DOI:** 10.3390/dj9020016

**Published:** 2021-01-31

**Authors:** Arkadiusz Dziedzic

**Affiliations:** Department of Restorative Dentistry with Endodontics, Medical University of Silesia, 40-055 Katowice, Poland; adziedzic@sum.edu.pl

In March 2020, dental care providers, including special care dentistry (SCD) services, had to face an extraordinary change of their standard operating procedures (SOP), with deferred domiciliary dental care, withholding conscious dental sedation services and disrupted routine care [1,2,3]. As the coronavirus disease COVID-19 (COVID-19) pandemic escalated further across the world, at the end of 2020 an entire ‘new reality’ has emerged affecting primary and secondary dental care services, shadowed by a mass-testing and a vulnerable groups-targeted anti-COVID-19 vaccination. Whilst hurdles associated with SCD during subsequent waves of COVID-19 continue, the inevitable adjustment and adaptation of dental services would allow sufficient healthcare provision for local communities. The prevailing increase of the public attention on telehealth, and, simultaneously observed, the lowered interest in any other aspects of care overall, was noted since the beginning of COVID-19 outbreak. Nowadays, we must carefully monitor the service capacity, staff availability, cleaning, and fallow time, and, as equally, safety measures, and being mindful of prophetic planning for the upcoming year.

Since the pandemic onset, dental teams have recuperated, evolved, and redefined their role, attempting to meet patients’ demands. We have had to embrace a whole set of new ‘technical’ abilities to become more adaptable users of modern technologies, getting to know telehealth and online consultation methods [4,5,6] which have become an integrated part of daily life. They seemingly enhanced our communication skills while providing a remote triaging or dealing with phobic and medically compromised individuals without pharmacological anxiety management measures [3]. Alas, ‘teledentisty’ might not be applicable in some SCD cases, when patient’s cooperation, understanding, and online competency is limited (dementia, learning disabilities, mental health problems). The global interest in phone or online consultations for health purposes appeared to have sharply increased in March 2020, and this trend maintains an adequate level at the end of 2020 (Figure 1).

The profound changes in work pattern took place recently within SCD, while dental teams were adopting urgent, emergency access dental care mode, resuming educational programs, and more importantly, preventing services from further debilitation [7,8]. Are we better prepared for a prolonged COVID-19 disruption? The answer is likely to be affirmative, although the expected adaptation and optimization process continues while the options remain limited, despite stabilizing our capacity of care provision. Being equipped with up-to-date guidelines, statutory regulations from public health authorities [9], and, equally, having support from joint commitment of professional bodies, such as International Association for Disability & Oral Health (IADH), Society for the Advancement of Anaesthesia in Dentistry (SAAD), British Society for Disability and Oral Health (BSDH), Royal Colleges of Surgeons, Dental Faculties (RCS), also by exchanging our knowledge during online courses, we are convinced that SCD acquired an effective defence against ‘disruptive healthcare’. The so-called ‘mimicry effect’ allows a self-adjustment to an unfavorable environment, including re-organization of SCD worldwide [10].

Predictably, there are yet various ‘no-win’ prone-to-fail critical situations, which cannot be resolved promptly. Care homes residents, patients with dementia who require domiciliary care, problems with ambulance transport for disabled individuals, deferred general anesthesia and conscious sedation for pre-cooperative young patients are only the fraction of examples of every-day practice challenges. These have been mitigated by a partial resumption of postponed operative care and resources within a secondary care level. The urgent need for wider promotion of conscious dental sedation (CDS) training has arisen during the pandemic [11], a direct result of suspended specialist services, including a shortage of personnel. One can notice that the general anesthesia provision appeared to decline sharply on a global scale as a result of the pandemic. Prompt implementation of ‘fast-track’ in-house dental training in conscious sedation techniques would inevitably de-escalate the growing need for general anesthesia provision in hospital setting, enabling the establishment of regional centres for CDS. This should be addressed in new SCD training curricula. There is also an opportunity to learn some techniques vitally important for intravenous sedation by, e.g., being involved in cannulation of hospitalized patients in case of deployment, to deal with COVID-19 crisis.

Worryingly, a vast proportion of patients with pre-existing conditions choose not to seek medical or dental care. This attitude might have been induced by ongoing anxiety and fear, pandemic uncertainty, mental health problem aggravation, and reluctance to attend dental appointments due to possible exposure, leading to an increased risk of COVID-19-related complications. This is in line with predictions associated with the ‘vicious circle’ of severe impact and health deterioration during a pandemic time [12]. From the public perspective, it should not be a surprise that worldwide Google Trends cumulative data revealed the decrease in online searching for such terms as ‘special care dentistry’ and ‘special dental care’ since the pandemic started, indicating a global healthcare disruption. As predicted, this data has reversed during the second part of 2020, with a gradually increasing, although still fluctuating, inclination (Figure 2).

With a prospect of mass vaccination against COVID-19, the resumption has been already commenced addressing the need to gradually re-establish routine preventative and operative dental care. Currently, the main dilemma appears related to returning to a pre-pandemic pattern of work or restricting dental care to a minimum, with a care planning focusing on achieving stabilization. The atraumatic, non-aerosol generating restorative treatment, wider use of silver diamine fluoride (SDF), and ‘ad hoc’ urgent dentistry might be adequate for efficient pain management as interim methods [1,13]. However, what would be a long-term impact of deferred routine treatment in more complex SCD cases? During care planning for persons who are, clinically, extremely vulnerable, the benefits of receiving dental treatment must be weighted up against risk of exposure and potential complications, and what is more, a shared decision is paramount [14].

Regardless of positive signs of SCD recovery, services remain in an uncertain position, trying to tackle primary dental problems, including uncomplete courses of treatment and waiting lists that accumulated exponentially. Nevertheless, the support from local authorities and commissioners has helped to re-organize the structure of SCD services. Considering the optimal management of patients with special needs, it would be prudent, although logistically difficult, to introduce point-of-care regular COVID-19 testing (either rapid molecular RT-qPCR or SARS-CoV-2 antigen tests) [15,16], i.e., weekly for institutionalized staff and in the form of pre-treatment check for patients.

Inevitably, not only persons with complex medical problems but also dental and medical professionals working on the ‘front line’, exposed to significant clinical challenge of this generation, would require specialist psychological counselling to deal with COVID-19 stress-related health problems [17]. As dental team members are exposed to aerosol generating procedure on regular basis, they have also a legitimate reason to be concerned about the wellbeing of their household members, who may belong to high-risk group of medically compromised persons. Despite the anticipated rapid, reliable testing (PCR/loop-mediated isothermal amplification LAMP/antigens tests) for staff and patients and already commenced vaccination against COVID-19, the essential arrangements defined by SOP regulations will maintain their primary role in protecting both dental teams and patients. It is debatable, whether the anti-COVID-19 vaccination and herd mass immunity could replace the need to use enhanced personal protective equipment for aerosol generating procedures in the future.

At the beginning of 2021, the summarizing points from the first Editorial remain valid and the same: lingering challenges, like currently occurring ones, make us more resilient, allowing a gradual improvement of care services. These constructive aspects will benefit our patients, who, I believe, may also teach us how to be adaptable and fit-for-purpose providers. We all have a sincere prediction that broadly executed anti COVID-19 vaccination program will accelerate the routine and specialist practice resumption.

## Figures and Tables

**Figure 1:**
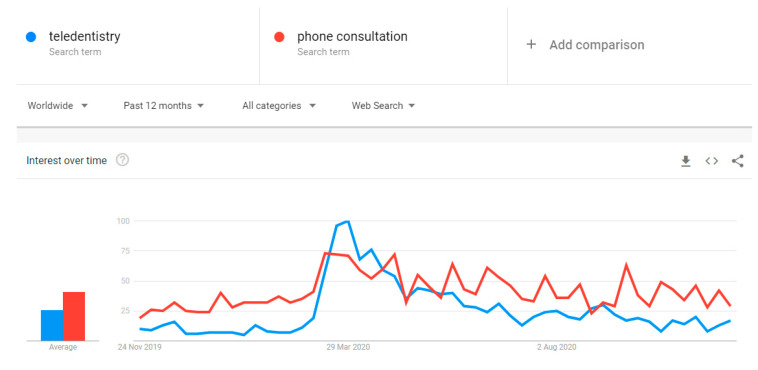
The worldwide online searching of terms ‘teledentistry’ and ‘phone consultation’ (Google Trends, accessed 14/11/2020) over the last 12 months.

**Figure 2:**
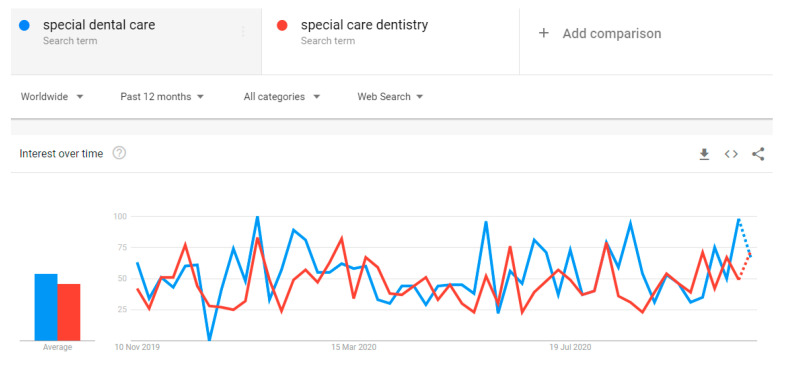
The worldwide online searching for ‘special dental care’ and ‘special care dentistry’ terms (data extracted from Google Trends, accessed 14/11/2020) over the last 12 months.

## Data Availability

Not applicable.
